# TCHP drives hepatocarcinogenesis through LLPS-mediated AURKA condensation and enables synergistic therapy

**DOI:** 10.1038/s41419-026-08681-6

**Published:** 2026-04-25

**Authors:** Jingshi Li, Yan Li, Xilang Pan, Shu Wang, Zaisheng Lin, Ruida He, Suning Wang, Xin Huang, Jia-Qi Li, Zhiwei Zhang, Liang Wang, Xiujuan Zhang, Xuxu Sun, Muqing Cao

**Affiliations:** 1 Precision Research Center for Refractory Diseases, Shanghai Jiao Tong University Pioneer Research Institute for Molecular and Cell Therapies, Shanghai General Hospital, Shanghai Jiao Tong University School of Medicine, State Key Laboratory of Innovative Immunotherapy, School of Pharmaceutical Sciences, Shanghai Jiao Tong University, Shanghai, China; Department of Pathophysiology, College of Basic Medical Sciences, Shanghai Jiao Tong University, Shanghai, China; 2 Department of Biochemistry and Molecular Cell Biology, State Key Laboratory of Systems Medicine for Cancer, Shanghai Key Laboratory for Tumor Microenvironment and Inflammation, Shanghai Jiao Tong University School of Medicine, Shanghai, China; 3 Department of Gastroenterology, Children’s Hospital of Soochow University, Suzhou, China; 4 Department of Oncology, Affiliated Hospital of Hebei University of Engineering, Handan, China; 5 School of Life Sciences, Jiangsu Normal University, Xuzhou, China

**Keywords:** Oncogenes, Cell division

## Abstract

Liver cancer is a leading cause of cancer-related death worldwide. Centrosomal proteins play critical roles in maintaining mitotic fidelity, and their dysregulation contributes to tumorigenesis. Trichoplein (TCHP), a centrosomal protein has recently been implicated in cell cycle progression, but its role in liver cancer remains unclear. In this study, we demonstrate that *TCHP* is markedly upregulated in hepatocellular carcinoma and hepatoblastoma and is associated with poor patient survival. Functional analyses revealed that *TCHP* overexpression accelerates hepatocarcinogenesis in mice, while its depletion suppresses tumor growth by inducing mitotic defects and extensive tumor cell death. Mechanistically, TCHP safeguards mitotic fidelity by localizing to centrosomes and promoting liquid–liquid phase separation–driven condensate formation with AURKA, thereby enhancing its activation. Importantly, TCHP inhibition not only suppresses tumor growth directly but also sensitizes liver cancer cells to the AURKA inhibitor alisertib, allowing tumor suppression at reduced drug doses and mitigating toxicity risks. Collectively, our findings establish *TCHP* as a potential oncogenic driver and therapeutic vulnerability in liver cancer and highlight the TCHP–AURKA axis as a promising target for synergistic treatment strategies.

## Introduction

Primary liver cancer represents a malignancy with high global incidence and mortality rates. Hepatocellular carcinoma (HCC) constitutes approximately 90% of primary liver cancers and has emerged as the third leading cause of cancer-related deaths worldwide [1, 2]. While recent advances in diagnostic imaging techniques and molecular profiling have improved early detection, and the development of targeted therapies (e.g., sorafenib, lenvatinib) and immunotherapies has expanded treatment options, the clinical outcomes for advanced HCC remain unsatisfactory. This therapeutic challenge stems primarily from the tumor’s remarkable heterogeneity, unclear intrinsic mechanisms, and chemoresistance, et.al [3–5]. These unmet clinical needs highlight the urgent imperative to identify novel molecular drivers of hepatocarcinogenesis and develop innovative combination therapeutic strategies that can simultaneously target multiple oncogenic pathways while minimizing systemic toxicity.

The centrosome serves as the microtubule-organizing center in most eukaryotic cells and plays a critical role in governing spindle orientation during mitosis and maintaining genome stability [6, 7]. Centrosome dysfunction disrupts spindle assembly, promotes chromosome missegregation, and drives genomic instability, thereby facilitating malignant transformation and tumor progression [8, 9]. Many conventional chemotherapeutic agents exploit this vulnerability by perturbing mitosis, leading to prolonged mitotic arrest and subsequent apoptosis. Moreover, inhibition of the spindle assembly checkpoint has been shown to sensitize certain chemotherapy-resistant cancer cells. Aurora a kinase (AURKA), a key centrosomal kinase, is indispensable for maintaining mitotic fidelity, and its activation is required for mitotic entry, centrosome maturation, centriole separation, and chromosome alignment [10–12]. Dysregulated expression or hyperactivation of AURKA has been implicated in a wide range of human cancers. Although selective AURKA inhibitors such as alisertib demonstrated potent anti-tumor efficacy in both preclinical and clinical studies, further clinical development was halted due to severe dose-limiting off-target toxicity [13, 14].

Trichoplein (TCHP) has been identified as a centrosomal protein that localizes to centrioles [15, 16]. It plays a crucial role in regulating primary cilium assembly by binding and activating AURKA, thereby suppressing aberrant ciliogenesis in proliferating cells. Loss of TCHP in RPE1 cells, a noncancerous cell line, triggers G_0_/G_1_ cell cycle arrest [16, 17]. Consistently, knockout of *Tchp* in mice results in ciliary elongation and suppression of the insulin/Akt signaling pathway, ultimately leading to restricted adipogenesis [18, 19]. Clinically, *TCHP* downregulation has been reported in 22% of bladder cancers and 23% of breast cancers [20]. These findings suggest that TCHP may play a broader role in tumor biology, yet beyond its known function in ciliogenesis, potentially involving other unidentified mechanisms.

Given its role in regulating cell proliferation, we investigated the contribution of TCHP to liver cancer pathogenesis. In this study, we demonstrate that TCHP drives hepatocarcinogenesis by promoting tumor cell proliferation possibly through liquid-liquid phase separation (LLPS) -mediated interaction with AURKA, facilitating its spindle localization and activation. Depletion of TCHP impaired AURKA function, suppressed cancer cell proliferation, and attenuated liver tumor development. Importantly, combined targeting of TCHP and AURKA exerted synergistic anti-tumor effects, enhancing the efficacy of AURKA inhibition, which may reduce the off-target toxicities associated with high-dose treatment. Our results identify TCHP as a key centrosomal regulator of liver cancer progression, and demonstrate the therapeutic potential of targeting TCHP-containing pathways in combination therapies.

## Results

### *TCHP* upregulation promotes hepatocarcinogenesis

To investigate the role of TCHP in cancer, we performed pan-cancer analysis of *TCHP* using patient data from The Cancer Genome Atlas (TCGA) database, among which liver hepatocellular carcinoma (LIHC) showed the most significant differential *TCHP* expression between tumor and adjacent normal tissues (Fig. S1A, B). Furthermore, subsequent survival analysis revealed that high *TCHP* expression correlated most significantly with poor prognosis in LIHC (Fig. S1C), indicating *TCHP* as a potential critical risk factor in LIHC.

**Fig. 1:**
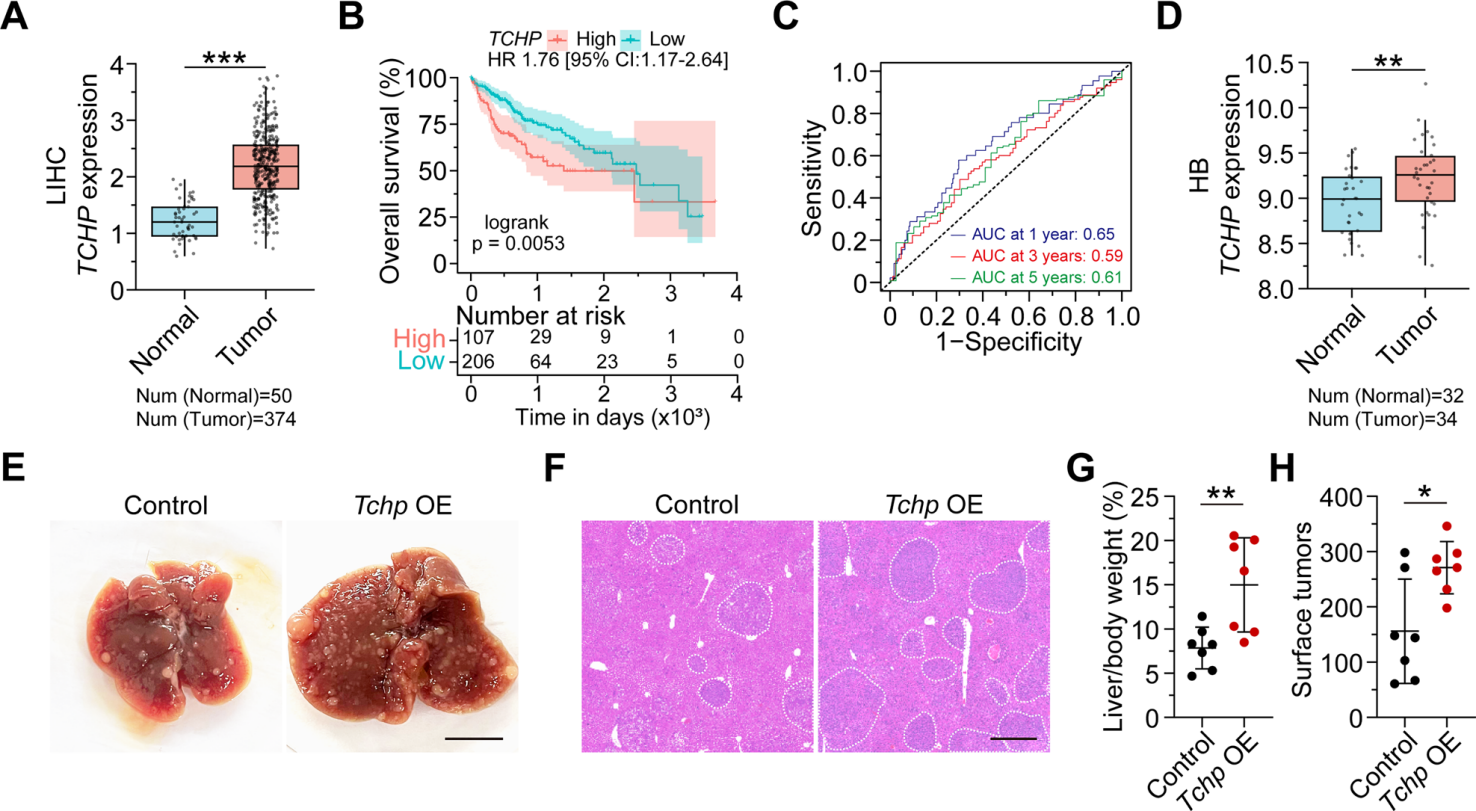
*TCHP* upregulation promotes hepatocarcinogenesis. **A** Comparison of *TCHP* expression between normal (*n* = 50) and tumor (*n* = 374) tissues in the TCGA-LIHC cohort. **B** Kaplan-Meier curves of overall survival based on *TCHP* expression in 444 LIHC patients (Log-rank test, hazard ratio = 1.76, *p* = 0.0053). **C** Receiver operating characteristic (ROC) curve analysis was performed to describe the accuracy of the scores for predicting 1-, 3-, and 5-year overall survival in the TCGA-LIHC cohort. **D** Comparison of *TCHP* expression between normal (*n* = 32) and tumor (*n* = 34) tissues in the R2-HB cohort. **E** Representative specimens of control and *Tchp*-overexpressed groups. Scale bar = 1 cm. F Representative H&E staining images of liver sections of control and *Tchp*-overexpressed mice. Scale bar = 500 μm. **G**,**H** Quantitative analysis of liver/body ratio (**G**) and tumor numbers (**H**) of control and *Tchp*-overexpressed mice. Data were presented as mean ± SD (*n* = 7; two-tailed unpaired Student’s *t* test).

We then evaluated *TCHP* expression levels in LIHC patient samples from TCGA database. *TCHP* expression was significantly elevated in tumor tissues compared with normal liver tissues (Fig. 1A). Critically, high *TCHP* expression was also associated with poor overall survival (OS) in LIHC patients (Fig. 1B, C). Gene expression analysis in the human hepatoblastoma (HB) cohorts (*n* = 66) from the online R2 genomics analysis and visualization platform similarly revealed increased *TCHP* expression in tumor tissues (Fig. 1D). Together, these results demonstrate aberrant *TCHP* expression in liver tumors and suggest its involvement in liver cancer pathogenesis.

To further assess the functional role of *TCHP* in liver cancer, we established in vivo liver cancer mouse model using hydrodynamic transfection method in wild-type C57BL/6 mice. Overexpression of *YAP* and *CTNNB1* induced liver tumor formation, as confirmed by gross morphology and H&E staining (Fig. 1E, F). Notably, concomitant *Tchp* overexpression significantly accelerated liver cancer progression, as evidenced by an increased liver/body weight ratio (Fig. 1G) and elevated surface tumor nodule counts compared to the controls (Fig. 1H). These in vivo evidences indicated that overexpression of *TCHP* promotes hepatocarcinogenesis.

### Downregulation of *TCHP* suppresses liver cancer progression

**Fig. 2:**
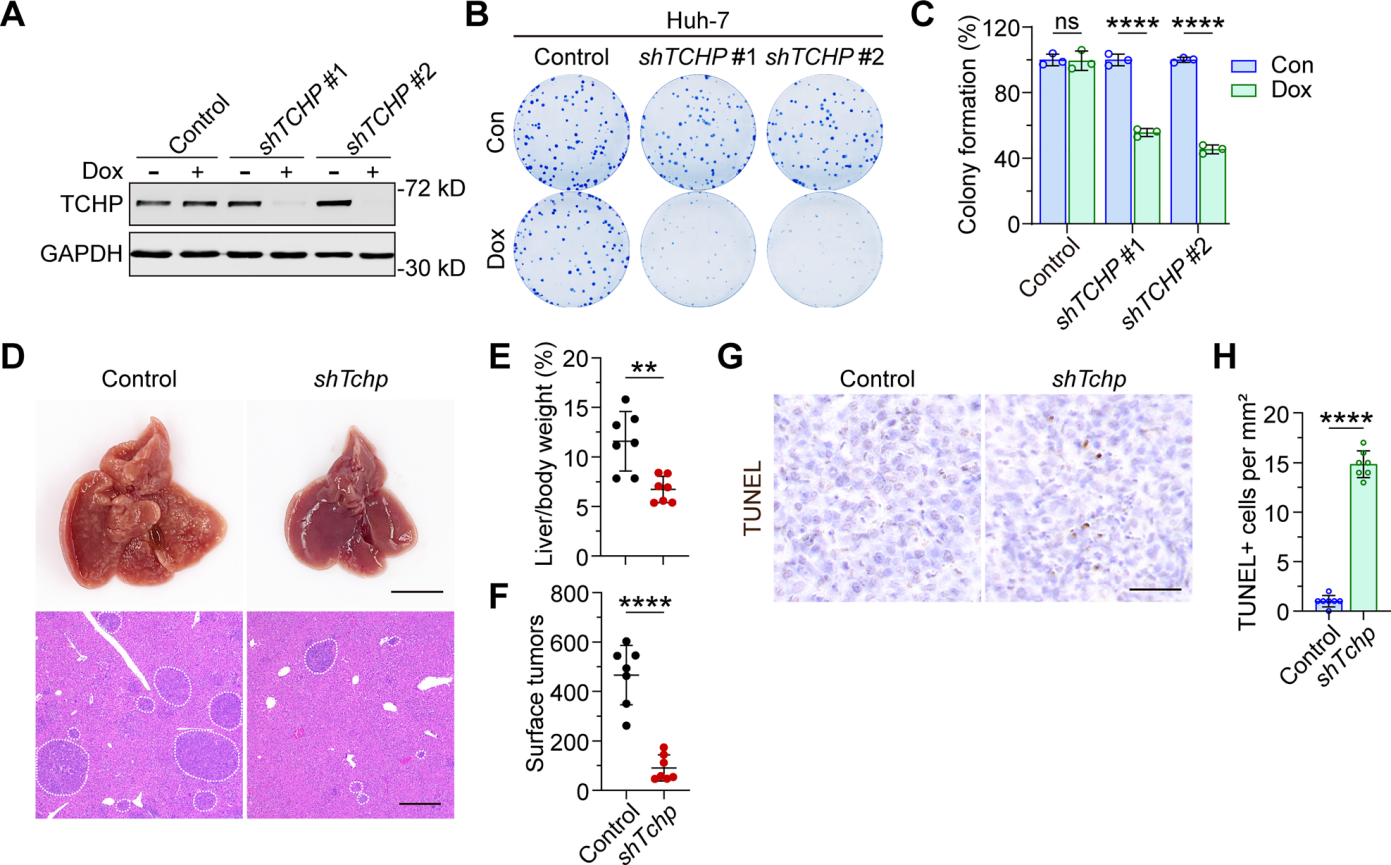
*TCHP* knockdown suppresses liver cancer progression. **A** Western blot analysis of TCHP levels before and after dox treatment in control and *TCHP*-knockdown Huh-7 cells. B After dox treatment, *TCHP*-knockdown Huh-7 cells showed decreased average colony numbers in the plate colony formation assay. **C** Quantification of colony counts in (**B**). Data were presented as mean ± SD (*n* = 3; two-tailed unpaired Student’s *t* test). **D** Representative specimens (top panels) and H&E staining images (lower panels) of control and *Tchp*-knockdown mice. Scale bar = 1 cm (top panels), 500 μm (lower panels). **E**,**F** Quantitative analysis of liver/body ratio (**E**) and tumor numbers (**F**) of control and *Tchp*-knockdown mice. Data were presented as mean ± SD (*n* = 7; two-tailed unpaired Student’s *t* test). G,H TUNEL staining of liver tissues from control (left panel) and *Tchp*-knockdown (right panel) mice. Scale bar = 50 μm. Data were presented as mean ± SD (*n* = 7; two-tailed unpaired Student’s *t* test).

Given the tumor-promoting effect of *TCHP* overexpression, we next examined whether its knockdown could suppress hepatocarcinogenesis. Colony formation assays showed significantly reduced colony counts in *TCHP*-deficient cells compared to controls (Fig. 2A–C), indicating impaired malignant potential. Furthermore, *TCHP* knockdown in the HepG2 cell line also led to reduced colony formation (Fig. S2A–C), further validating our conclusion that downregulation of *TCHP* impaired malignant potential in liver cancer cells.

In vivo, *Thcp* knockdown markedly reduced tumor burden in the liver cancer mouse model (Fig. 2D–F). Moreover, terminal deoxynucleotidyl transferase biotin-dUTP nick end labelling (TUNEL) staining revealed increased apoptosis in the *Tchp*-deficient tumors (Fig. 2G, H), suggesting that cell death contributes to the observed tumor suppression.

### Loss of TCHP induces mitotic defects in liver cancer cells

**Fig. 3:**
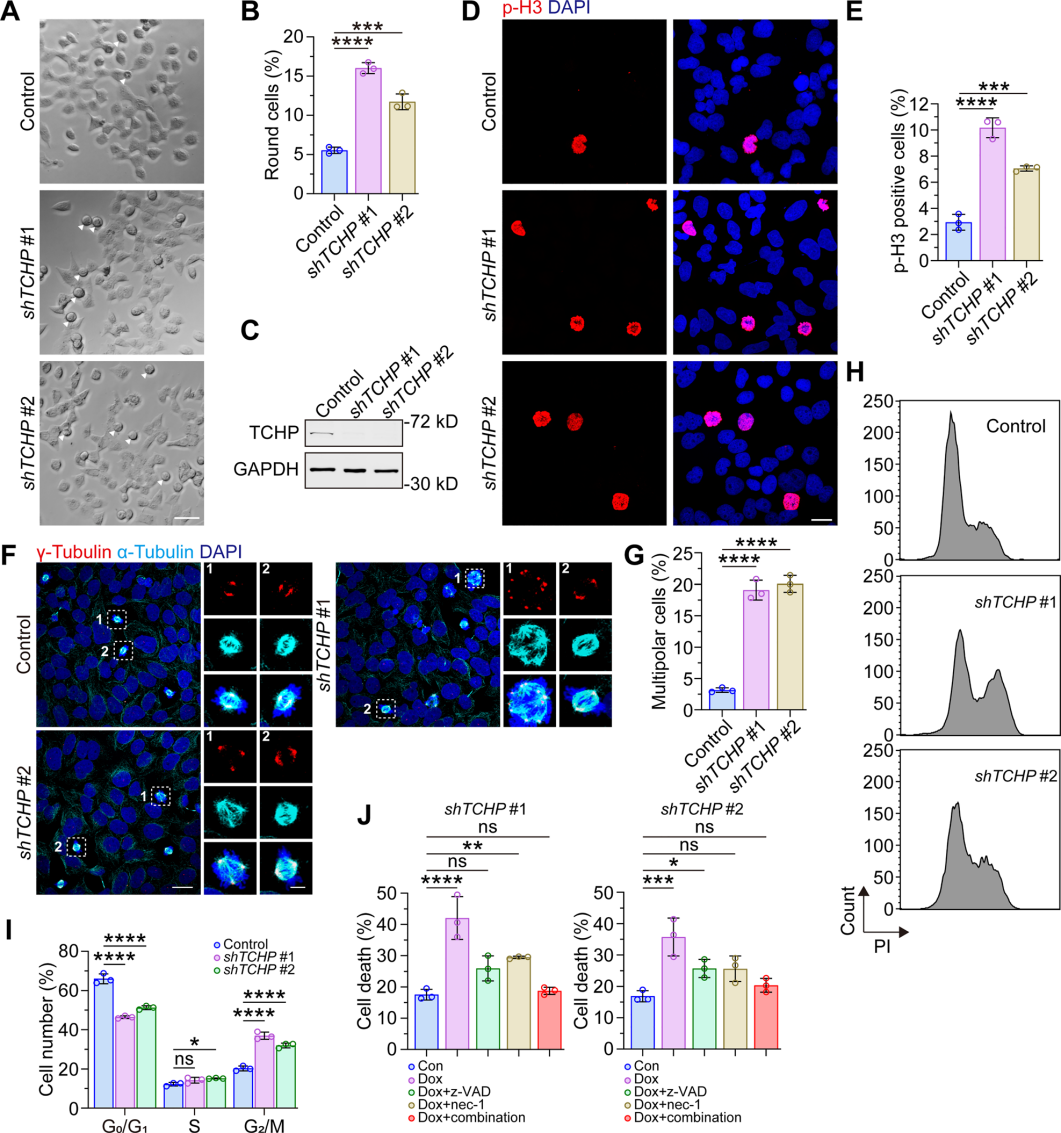
*TCHP* knockdown induces mitotic defects in liver cancer cells. **A** Phase contrast images of control and *TCHP*-knockdown Huh-7 cells in the presence of 500 ng/mL dox. Scale bar = 50 μm. **B** Quantification of round cells in (**A**). Data were presented as mean ± SD (*n* = 3; one-way ANOVA with Dunnett’s multiple comparisons test). **C** Western blot analysis of TCHP levels in control and *TCHP*-knockdown Huh-7 cells after dox treatment. **D** Representative immunofluorescence images of p-H3 (red) and DAPI (blue) in control and *TCHP*-knockdown Huh-7 cells. Scale bar = 20 μm. **E** Quantification of p-H3 positive cells in (**D**). Data were presented as mean ± SD (*n* = 3; one-way ANOVA with Dunnett’s multiple comparisons test). **F**,**G** Representative immunofluorescence images and quantitative analysis of multipolar cells demonstrated abnormal amplification of centrosomes in *TCHP*-knockdown Huh-7 cells compared to the control group. γ-tubulin (red), α-tubulin (cyan) and DAPI (blue). Scale bar = 20 μm (left), 5 μm (right). Data were presented as mean ± SD (*n* = 3; one-way ANOVA with Dunnett’s multiple comparisons test). **H** DNA content analysis using flow cytometry in control and *TCHP*-knockdown Huh-7 cells. **I** Quantification analysis of cell percentage in each phase in control and *TCHP*-knockdown Huh-7 cells. Data were presented as mean ± SD (*n* = 3; one-way ANOVA with Dunnett’s multiple comparisons test). **J** Cell death rate in *TCHP*-knockdown Huh-7 cells after 20 μM z-VAD, 20 μM nec-1 and combination treatment. Data were presented as mean ± SD (*n* = 3; one-way ANOVA with Dunnett’s multiple comparisons test).

In addition to suppressing cancer cell colony formation, we noticed that knocking down of *TCHP* induced marked morphological alterations in liver cancer cells. Phase-contrast microscopy revealed a significant increase in round cells in *TCHP*-deficient cells (Fig. 3A), resembling mitotic morphology. Quantitative analysis confirmed elevated proportions of round cells in *TCHP* knockdown cells (Fig. 3B, C). To further assess mitotic progression, we performed phospho-histone H3 (Ser10) (p-H3) staining in *TCHP*-deficient cells. The staining demonstrated an increased mitotic index in *TCHP*-deficient cells (Fig. 3D, E).

Despite the accumulation of mitotic cells, depletion of TCHP induced severe spindle assembly defects. Co-staining of α-tubulin and γ-tubulin revealed a pronounced multipolar spindle phenotype (Fig. 3F, G). Further DNA content analysis via flow cytometry indicated the significant G_2_/M phase accumulation in TCHP-depleted cells (Fig. 3H, I). Together with impaired colony formation and enhanced cell death observed in vivo (Fig. 2A, G), these findings suggest that cells entering mitosis without TCHP may experience lethal mitotic defects, finally affecting cell viability.

Given the observed mitotic defects, we next investigated the contribution of cell death pathways to this phenotype. As measured by flow cytometry, treatment of *TCHP*-deficient cells with the apoptosis inhibitor z-VAD or the necroptosis inhibitor necrostatin-1 (Nec-1) only partially inhibited cell death, while combination treatment reached near-complete restoration (Fig. S3A and Fig. 3J). These results indicated that TCHP depletion induces a mixed cell death response involving both apoptosis and necroptosis. Collectively, our results indicated that loss of *TCHP* induces mitotic defects, leading to cell death in liver cancer cells.

Previous studies demonstrated that TCHP depletion in RPE1 cells induces the G_0_/G_1_ cell cycle arrest with the assembly of primary cilia [16]. To test whether similar effects occur in liver cancer cells, we performed Ki67 immunostaining in Huh-7 cells. However, the staining revealed no significant change in the proportion of Ki67-positive cells between *TCHP*-knockdown and control cells (Fig. S4A, B), suggesting that TCHP depletion does not induce cell cycle arrest at G_0_/G_1_ phase in the Huh-7 cells. We further assessed cilia assembly in TCHP-depleted Huh-7 cells under serum-starved and non-starved conditions. Consistent with published research [21], immunofluorescence analysis confirmed the absence of cilia in non-starved Huh-7 cells, and fewer than 1% of cells displayed cilia upon serum starvation, regardless of *TCHP* knockdown status (Fig. S4C–E). Collectively, these results demonstrate that in liver cancer cells, TCHP depletion impairs proliferation through mitotic defects and subsequent cell death, independent of ciliogenesis or G_0_/G_1_ cell cycle arrest.

### TCHP regulates spindle localization and activation of AURKA through LLPS

Since *TCHP* knockdown in Huh-7 cells led to mitotic defects and cell death phenotype resembling those observed with *AURKA* depletion [22–24], and given previous evidence that TCHP directly binds and activates AURKA in G_1_/S transition [16], we investigated whether loss of TCHP induces mitotic abnormalities and cell death by impairing AURKA activation and spindle assembly before M phase.

**Fig. 4:**
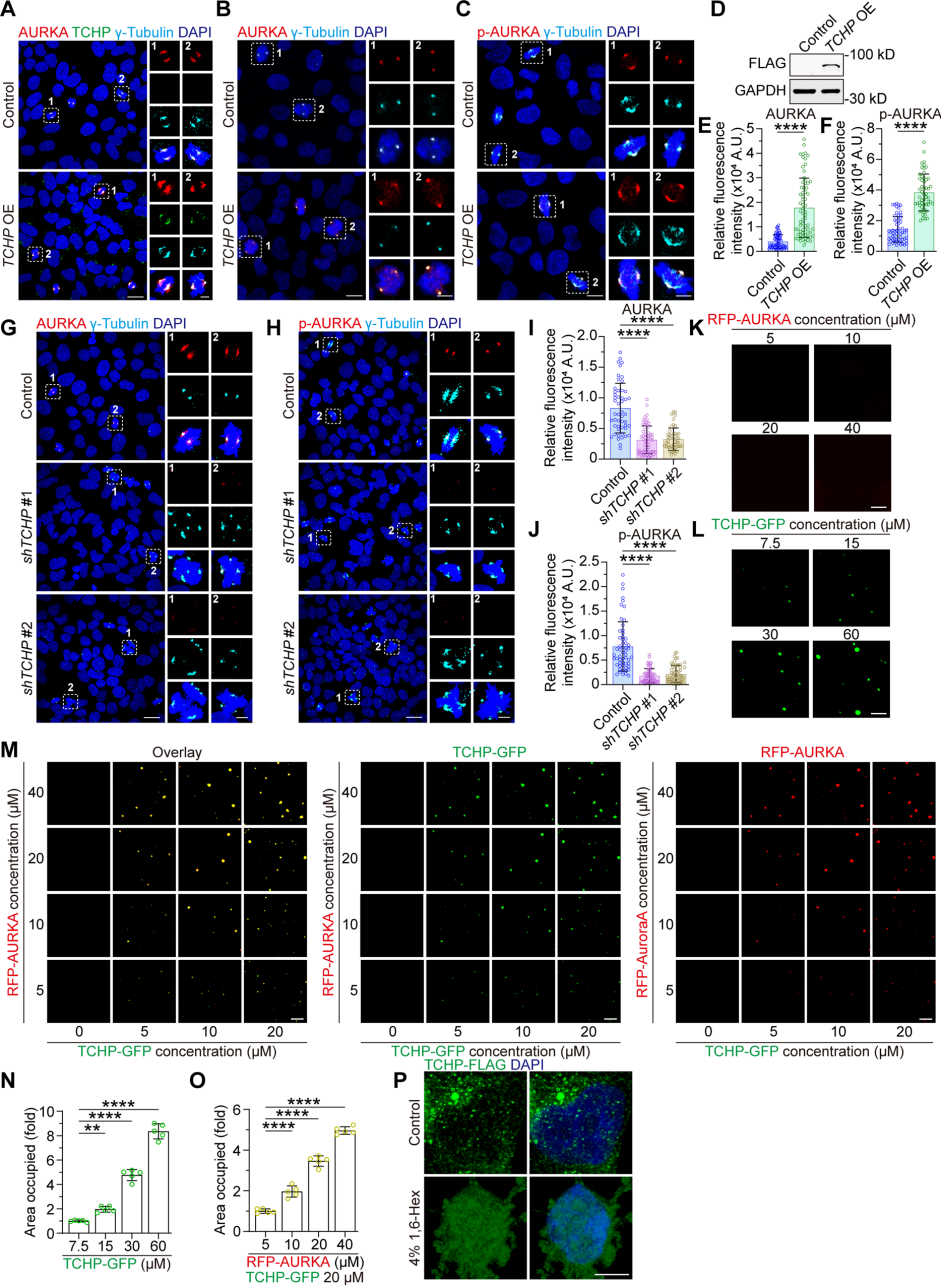
TCHP regulates spindle localization and activation of AURKA through LLPS. **A** Representative immunofluorescence images of AURKA (red), TCHP (green), γ-tubulin (cyan) and DAPI (blue) in control and *TCHP*-overexpressed Huh-7 cells. Scale bar = 20 μm (left), 5 μm (right). **B** Representative immunofluorescence images of control and *TCHP*-overexpressed Huh-7 cells stained with AURKA (red), γ-tubulin (cyan) and DAPI (blue). Scale bar = 10 μm (left), 5 μm (right). **C** Representative immunofluorescence images of p-AURKA (red), γ-tubulin (cyan) and DAPI (blue) in control and *TCHP*-overexpressed Huh-7 cells. Scale bar = 10 μm (left), 5 μm (right). **D** Western blot analysis of TCHP levels in control and *TCHP*-overexpressed Huh-7 cells in the presence of dox (500 ng/mL). (E, F) Relative fluorescence intensity quantification of AURKA (**E**) and p-AURKA (**F**) in (**B**) and (**C**). Data were presented as mean ± SD (average of 50 cells per group; two-tailed unpaired Student’s *t* test). **G** Representative immunofluorescence images of control and *TCHP*-knockdown Huh-7 cells stained with AURKA (red), γ-tubulin (cyan) and DAPI (blue). Scale bar = 20 μm (left), 5 μm (right). **H** Representative immunofluorescence images of p-AURKA (red), γ-tubulin (cyan) and DAPI (blue) in control and *TCHP*-knockdown Huh-7 cells. Scale bar = 20 μm (left), 5 μm (right). **I**,**J** Relative fluorescence intensity quantification of AURKA (**I**) and p-AURKA (**J**) in (**G**) and (**H**). Data were presented as mean ± SD (average of 50 cells per group; one-way ANOVA with Dunnett’s multiple comparisons test). **K**,**L** Images of RFP-AURKA (**K**) and TCHP-GFP (**L**) droplet formation at 37 °C with indicated concentrations separately. Scale bar = 5 μm. **M** Purified TCHP-GFP was incubated to allow coacervation in the presence of purified RFP-AURKA. Scale bar = 10 μm. **N** Quantitative analysis of the droplet formation of TCHP-GFP in (**L**). Data were presented as mean ± SD (*n* = 5; one-way ANOVA with Dunnett’s multiple comparisons test). **O** Quantitative analysis of the droplet formation of TCHP-GFP and RFP-AURKA when TCHP-GFP is at 20 μM concentration. Data were presented as mean ± SD (*n* = 5; one-way ANOVA with Dunnett’s multiple comparisons test). **P** Representative immunofluorescence images of TCHP-FLAG (green) and DAPI (blue) in 4% 1,6-hexanediol treated *TCHP*-overexpressed Huh-7 cells compared to non-treated *TCHP*-overexpressed Huh-7 cells. Scale bar = 5 μm.

To test this, we generated Tet-ON Huh-7 cells expressing GFP- and Flag-tagged TCHP in a doxycycline (Dox)-dependent manner. TCHP, a protein binding keratin filaments or mitochondria [25, 26], was shown to localize at both mother and daughter centrioles [15, 16]. Immunofluorescence confirmed the centrosomal localization of TCHP, where it localized with AURKA and γ-tubulin but not with the mitochondrial marker TOM20 in Huh-7 cells (Fig. S5A and Fig. 4A). Overexpression of TCHP enhanced both AURKA spindle localization and autophosphorylation at Thr288 (p-AURKA) (Fig. 4B–F). Conversely, *TCHP* knockdown impaired AURKA localization and activation at the spindle poles (Fig. 4G–J). Moreover, *TCHP* knockdown in HepG2 cells likewise resulted in an increased mitotic index and impaired spindle localization and activation of AURKA (Fig. S6A–F). These findings indicate that TCHP facilitates AURKA recruitment to the spindle and promotes its activation.

Given that AURKA activation has previously been shown to be regulated through BuGZ-mediated biomolecular condensates [27], we investigated whether TCHP exerts a similar mechanism. We purified GFP-tagged full-length TCHP (TCHP-GFP) and RFP-tagged full-length AURKA (RFP-AURKA). Consistent with previous studies, in vitro assays revealed that RFP-AURKA remained diffuse when incubated alone (Fig. 4K), whereas GFP-TCHP spontaneously formed liquid-like droplets (Fig. 4L). When mixed together, RFP-AURKA was selectively enriched within TCHP condensates and, in turn, promoted TCHP phase separation (Fig. 4M–O). These results demonstrate that TCHP drives LLPS with AURKA to form AURKA-enriched biomolecular condensates in vitro. To further explore biophysical evidence in vivo, we treated *TCHP*-overexpressed cells with 1,6-hexanediol [28], which dissolves the droplets formed by TCHP (Fig. 4P).

Taken together, these results demonstrated that TCHP interacts with AURKA and regulates its spindle localization and activation probably via LLPS, potentially linking TCHP to cancer-relevant mitotic regulation.

### Co-expression of *TCHP* and *AURKA* predicts poor prognosis in liver cancer

**Fig. 5:**
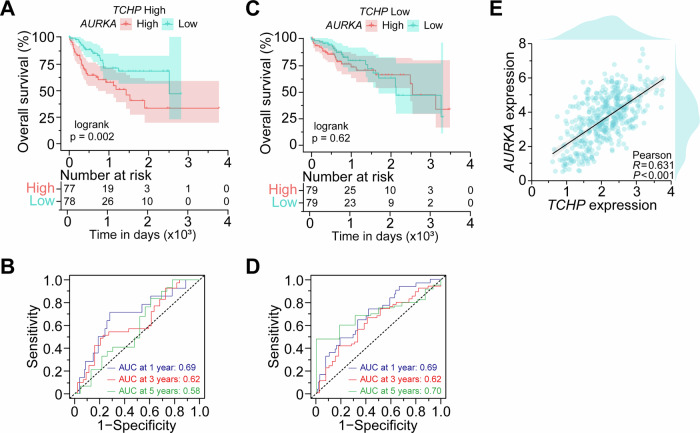
Co-expression of *TCHP* and *AURKA* predicts poor prognosis in liver cancer. **A–D** Cox proportional hazards analysis was performed to evaluate the combined effect of *TCHP* and *AURKA* expression on the overall survival of LIHC patient cohort from TCGA repository (**A**,**C**). ROC analysis was performed to describe the accuracy of the scores for predicting 1-, 3-, and 5-year overall survival in TCGA-LIHC patients (**B**,**D**). **E** The correlation analysis of *TCHP* and *AURKA* expression in the TCGA-LIHC cohort (*n* = 444).

To assess the clinical relevance of the TCHP-AURKA interaction, we examined *AURKA* expression in the human LIHC and HB cohorts. Consistent with *TCHP*, *AURKA* was upregulated in tumor tissues and was correlated with poor prognosis in LIHC and HB patients (Fig. S7A–D). Univariate and multivariate Cox regression revealed that while both *TCHP* and *AURKA* exhibited statistically significant associations with poor prognosis in the univariate Cox regression models, only *AURKA* retained the association in the multivariate model (Fig. S8A, B). This attenuation of *TCHP*’s prognostic significance upon adjustment for *AURKA* suggests the covariance between the two genes. Cox proportional hazards analysis showed that high *AURKA* expression predicted poor overall survival only in *TCHP*-high patients, but not in *TCHP*-low patients (Fig. 5A–D). Consistently, high *TCHP* expression predicted poor prognosis exclusively in *AURKA*-high LIHC patients, with no significant impact in *AURKA*-low patients (Fig. S7E–H). Moreover, correlation analysis further revealed a positive association between *TCHP* and *AURKA* expression in the human LIHC cohort (Fig. 5E). Notably, patients with concurrent high expression of both *TCHP* and *AURKA* showed the poorest survival (Fig. 5A), strongly suggesting their synergistic role in liver cancer progression.

### TCHP inhibition enhances the efficacy of AURKA inhibitor alisertib

**Fig. 6:**
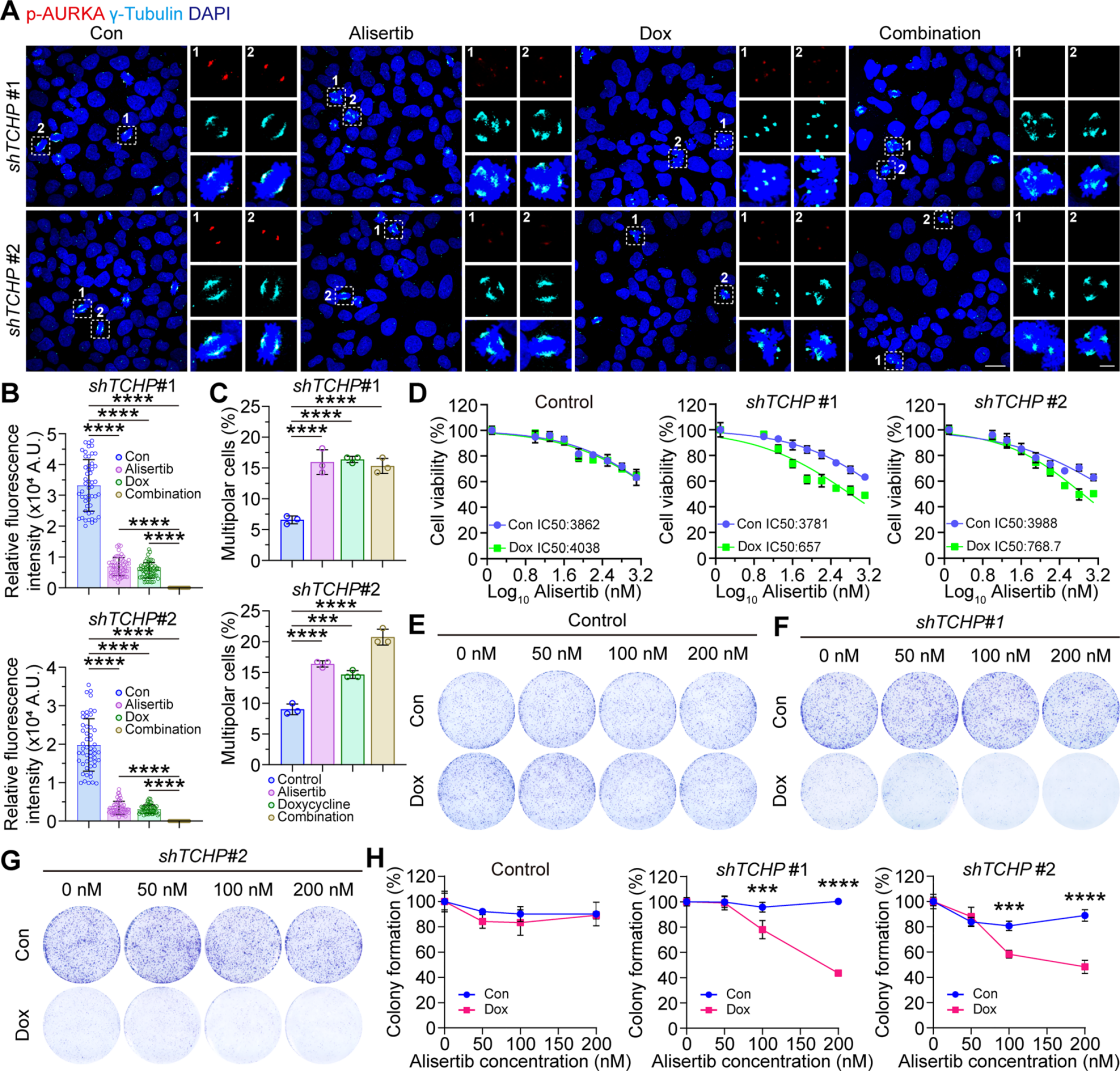
TCHP inhibition enhances the efficacy of AURKA inhibitor alisertib. **A** Representative immunofluorescence images of p-AURKA (red), γ-tubulin (cyan) and DAPI (blue) in *TCHP*-knockdown Huh-7 cells upon different treatments. Scale bar = 20 μm (left), 5 μm (right). **B** Relative fluorescence intensity quantification of p-AURKA in (**A**). Data were presented as mean ± SD (average of 50 cells per group; one-way ANOVA with Tukey’s multiple comparisons test). **C** Quantitative analysis of multipolar cells in *TCHP*-knockdown Huh-7 cells. Data were presented as mean ± SD (*n* = 3; one-way ANOVA with Dunnett’s multiple comparisons test). **D** CTG luminescence assay in control and *TCHP*-knockdown Huh-7 cells in the presence of alisertib and dox (500 ng/mL) showed reduced alisertib IC50 in *TCHP*-deficient cells. **E–G** Colony formation assay showed synergistic response to alisertib combined with *TCHP*-knockdown in Huh-7 cells at different alisertib concentrations. **H** Quantification of colony counts in (**E–G**) at different alisertib concentrations. Data were presented as mean ± SD (*n* = 3; two-way ANOVA with Sidak’s multiple comparisons test).

Given the functional interdependence, we investigated whether dual inhibition could more effectively suppress tumor cell proliferation. *TCHP* knockdown or treatment with the selective AURKA inhibitor MLN8237 (alisertib) each partially reduced AURKA phosphorylation, whereas the combination nearly abolished AURKA activation (Fig. 6A–C). These findings establish dual inhibition of TCHP and AURKA as a possible synergistic therapeutic strategy for liver cancer. Although alisertib showed antitumor efficacy in clinical trials [13, 14], its development was hindered by dose-limiting off-target toxicity. We therefore asked whether TCHP inhibition could potentiate the anti-tumor effects of low-dose alisertib while minimizing toxicity. Indeed, CellTiter-Glo (CTG) luminescence assay demonstrated that *TCHP* knockdown lowered the half maximal inhibitory concentration (IC50) of alisertib from 3780 nM to 657 nM, indicating that TCHP inhibition may enable therapeutic efficacy at reduced alisertib dose (Fig. 6D). Colony formation assay also exhibited enhanced effects at lower alisertib concentrations combined with *TCHP*-knockdown (Fig. 6E–H).

### Combined targeting of TCHP and AURKA enhances tumor suppression

**Fig. 7:**
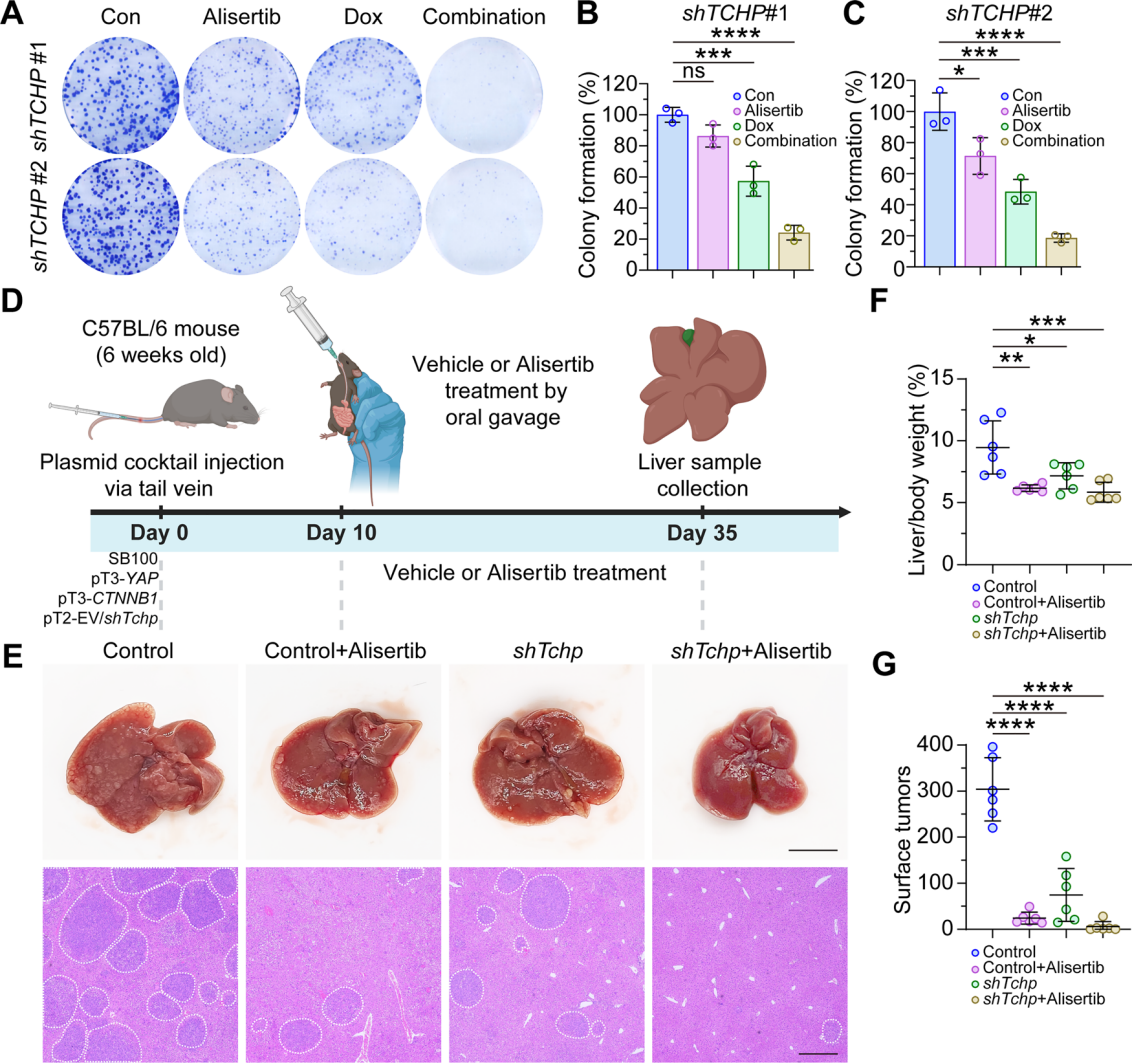
Combined targeting of TCHP and AURKA enhances tumor suppression. **A** Colony formation assay showed synergistic response to alisertib combined with *TCHP*-knockdown in Huh-7 cells. **B**,**C** Quantification of colony counts in (**A**). Data were presented as mean ± SD (*n* = 3; one-way ANOVA with Dunnett’s multiple comparisons test). **D** Schematic protocol of synergistic effect test in vivo. Created in https://BioRender.com. **E** Representative specimens (top panels) and H&E staining images (lower panels) of drug-treated tumor-bearing mice. Scale bar = 1 cm (top panels), 200 μm (lower panels). **F**,**G** Quantitative analysis of liver/body ratio (**F**) and tumor numbers (**G**) of drug-treated tumor-bearing mice. Data were presented as mean ± SD (*n* = 6; one-way ANOVA with Dunnett’s multiple comparisons test).

Colony formation assays further demonstrated that loss of TCHP enhanced the inhibitory effect of alisertib on cancer cell proliferation (Fig. 7A–C). Together, these findings indicate that TCHP depletion sensitizes tumor cells to AURKA inhibition, thereby enhancing therapeutic efficacy while potentially reducing drug-associated toxicity.

To further validate this strategy in vivo, we performed the combined inhibition of TCHP and AURKA (Fig. 7D). The knockdown efficiency of shRNAs targeting human *TCHP* and mouse *Tchp* was validated by qPCR (Fig. S9A, B). Consistent with the in vitro results, the combination therapy achieved significantly greater tumor suppression than either monotherapy (Fig. 7E–G), establishing TCHP as both a novel therapeutic target and a promising sensitizer for alisertib-based liver cancer treatment.

## Discussion

In this study, we identified a significant association between the centrosomal protein TCHP and liver cancer pathogenesis through analysis of LIHC and HB patient cohorts, and demonstrated the potential of TCHP as a therapeutic target in liver cancer. Functional studies showed that TCHP depletion suppresses hepatocarcinogenesis. This effect is mediated through impaired spindle localization and activation of the downstream kinase AURKA, thereby leading to cell death in cancer cells. Mechanistically, we uncovered that TCHP and AURKA co-assemble into LLPS-driven condensates, providing a novel mechanism for regulating AURKA activation and mitotic fidelity. Importantly, combined targeting of TCHP and AURKA with alisertib yielded synergistic anti-tumor efficacy in both cell and mouse models, offering preclinical evidence that the TCHP-AURKA axis represents a promising combinatorial therapeutic strategy for liver cancer.

Our findings also highlight cell-type-specific differences in TCHP function. Previous studies in RPE1 cells, a non-transformed cell line, reported that *TCHP* knockdown disrupts AURKA activation during G_1_ phase, induces primary cilium assembly, and causes G_0_/G_1_ cell cycle arrest [16]. In contrast, we observed that TCHP depletion in Huh-7 cells did not significantly increase the proportion of G_0_ phase cells, nor did it enhance ciliogenesis. Instead, loss of TCHP impaired AURKA activation during M phase, leading to mitotic multipolarity, prolonged M phase arrest, and extensive cell death. Consistently, depletion of TCHP in SV40 T-antigen transformed RPE1 cells failed to induce G_0_/G_1_ cell cycle arrest [17]. These divergent outcomes suggest that the role of TCHP is context-dependent, with its regulatory mechanisms varying across normal and cancerous cell types. Interestingly, previous studies in bladder and breast cancer reported that ectopic expression of *TCHP* reduced cell growth, suggesting its tumor-suppressive role [20]. This appears contradictory to our finding in liver cancer, where *TCHP* functions as an oncogenic driver. Notably, primary cilia are ubiquitously observed in prostate and breast tissues, and primary cilia have been shown to exert both pro- and anti-tumorigenic effects through Hedgehog (Hh) signaling, depending on the oncogenic context [29, 30]. Moreover, unlike prostate and breast cancers, liver cancer tissues universally lack primary cilia except in cholangiocytes [31], which suggests that TCHP likely promotes hepatocarcinogenesis via cilia-independent mechanisms. Taken together, these observations reconcile previous discrepancies and support the conclusion that *TCHP* functions as a tumor promoter in liver cancer.

Consistent with earlier biochemical studies showing that the N-terminal region of TCHP directly binds AURKA [16], our study extends these observations by demonstrating that TCHP promotes the assembly of AURKA-containing condensates, thereby enhancing its activation at centrosomes. While the structural determinants of this interaction remain to be fully defined, our results provide important mechanistic insights into how TCHP safeguards mitotic fidelity.

Alisertib, a selective Aurora kinase inhibitor with predominant activity against AURKA, has shown encouraging anti-tumor effects in early-phase clinical trials for multiple cancers. However, its clinical development was hampered by its significant dose-limiting off-target toxicity [13, 14]. Our findings suggest that targeting TCHP provides a rational upstream strategy to overcome this limitation. By disrupting TCHP, AURKA activation is attenuated, thereby sensitizing liver cancer cells to alisertib and enabling therapeutic efficacy at lower drug concentrations. This vertical inhibition approach not only enhances anti-tumor efficacy but also offers the potential to reduce systemic toxicity. Nevertheless, a comprehensive evaluation of this synergistic strategy across diverse preclinical models will be required to assess its efficacy and safety before clinical translation. Together, our study establishes TCHP as a potential oncogenic driver and highlights the TCHP-AURKA axis as a promising therapeutic vulnerability in liver cancer.

## Materials and methods

### Gene expression and survival analysis

The gene expression data and the corresponding clinicopathological characteristics of LIHC patients were downloaded from the TCGA data portal (https://gdc-portal.nci.nih.gov/) (accessed on 17th of February 2025). Additionally, HB patient gene expression data were obtained from the online R2 genomics analysis and visualization platform (http://r2.amc.nl). For survival analysis, OS rates were calculated via the Kaplan‒Meier method and compared via the log-rank test, which was generated using the “Survival” package, and the hazard ratio (HR) and 95% confidence intervals were calculated. Patients with LIHC were classified into high-expression and low-expression groups based on the median expression (Kmm) or the best cutoff expression (Kmb) of the selected genes. And the “timeROC” package was used to construct the time-dependent ROC curves. We also used univariate and multivariate COX regression analyses to validate the relationship between TCHP, AURKA and HCC prognosis. The analyses were conducted using R program (v4.4.1). Notably, due to insufficient patient follow-up data, the association between *TCHP* expression and OS in HB patients could not be definitively established.

### Animal experiments

C57BL/6 mice were purchased from Shanghai SLAC Laboratory Animal Co., Ltd. All animal experiments were approved by the Institutional Animal Care and Use Committees of Shanghai Jiao Tong University School of Medicine, Shanghai, China (Permit number: A-2022-014).

Plasmids for hydrodynamic tail vein injection were prepared using the EndoFree Mini plasmid Kit II (DP118, TIANGEN BIOTECH, Beijing).

Six-week-old male wild-type C57BL/6 mice were randomly allocated to experimental groups. For establishing liver cancer mouse models, we performed hydrodynamic tail vein injection of plasmid cocktail into wild-type C57BL/6 mice [32, 33]. Plasmids were diluted in physiological saline at a final volume of 10% mouse body weight. The plasmid ratio for *Tchp*-overexpressing group was pT3-*YAP*: pT3-*CTNNB1*: SB100: pT3-EV/pT3-*Tchp* (5 μg:5 μg:4 μg:10 μg), while in *Tchp*-knockdown group was pT3-*YAP*: pT3-*CTNNB1*: SB100: pT2-EV/pT2-*shTchp* (5 μg:5 μg:4 μg:10 μg). The cocktails were then administered via tail vein injection within 10 seconds to establish liver cancer mouse models. Liver tissues were harvested 5 weeks after transfection for further analysis. Notably, to ensure robust *Tchp* knockdown in mice, we utilized a pool of four distinct short hairpin RNA plasmids targeting *Tchp*.

For alisertib treatment in vivo, mice were treated with 15 mg/kg alisertib once a day for 6 days, and then treatment was stopped for 1 day. The drug was dissolved in 10% 2-hydroxypropyl-β-cyclodextrin-1% sodium bicarbonate and was administered by oral gavage. Vehicle or alisertib treatment was initiated 10 days after transfection, and the total duration was 25 days.

### Western blot analysis

Cell lysates were harvested with sodium dodecyl sulfate (SDS) sample buffer, and proteins were then loaded onto SDS-polyacrylamide gels and transferred to 0.2 µm NT nitrocellulose membranes (66485; BIOTRACE). After blocking with 5% milk, membranes were incubated with appropriate antibodies and detected by enhanced chemiluminescence or fluorescence-based imaging.

Antibodies used were: rabbit anti-TCHP (1:5000, 25931-1-AP, Proteintech), mouse anti-GAPDH (1:200,000, 60,004-1-Ig, Proteintech), mouse anti-FLAG (1:5000, F1804, Sigma-Aldrich), Peroxidase-conjugated AffiniPure Goat Anti-Rabbit lgG (H + L) (1:10,000, 111-035-003, Jackson Immunoresearch), Peroxidase-conjugated AffiniPure Goat Anti-Mouse lgG (H + L) (1:10,000, 115-035-003, Jackson Immunoresearch), IRDye^®^ 800CW Goat anti-Mouse IgG Secondary Antibody (1:20,000, 926-32210, LI-COR), IRDye^®^ 680RD Goat anti-Rabbit IgG Secondary Antibody (1:20,000, 926-68,071, LI-COR).

### Immunohistochemistry

Liver tissues were fixed by immersion in 4% PFA for 24 h and subsequently embedded in paraffin. For haematoxylin & eosin staining (H&E staining), the tissue section was treated with haematoxylin & eosin. Terminal deoxynucleotidyl transferase dUTP nick end labeling (TUNEL) assay was performed using TUNEL BrightGreen Apoptosis Detection Kit (A112, Vazyme).

### Immunofluorescence

Cells on coverslips were gently washed with phosphate-buffered saline (PBS) and then fixed with 4% paraformaldehyde (PFA) for 10 min, followed by permeabilization with pre-chilled methanol at −20 °C for 10 min. Following fixation, coverslips were blocked in 4% BSA (w/v), 0.5% Triton X-100 (v/v) in PBS, and all antibody incubation steps were carried out for 1 h at room temperature. Primary and secondary antibody dilutions were diluted in blocking buffer, with secondary staining being conducted in the dark. DNA was stained using DAPI. Immunofluorescence imaging was performed on a confocal microscope (FV3000, Olympus) with a 40× oil-immersion objective. For relative fluorescence intensity quantification, the average of 50 cells per group was calculated, and the fluorescence intensity of each cell was analyzed using ImageJ software [34].

Antibodies used were: mouse anti-α-tubulin (1:500, 66031-1-Ig, Proteintech), rabbit anti-Ki67 (1:400, ab15580, Abcam), mouse anti-ARL13B (1:1000, 75-287, NeuroMab), rabbit anti-TOM20 (1:500, sc-11415, Santa Cruz Biotechnology), rabbit anti-γ-tubulin (1:1500, T5192, Sigma-Aldrich), rabbit anti-Phospho-Histone H3-S10 (1:150, AP0002, ABclonal), mouse anti-γ-tubulin (1:1500, T5326, Sigma-Aldrich), mouse anti-AURKA (1:1500, 12100S, CST), rabbit anti-Phospho-AURKA (Thr288) (1:250, 2914S, CST), GFP Nanobody booster Alexa Fluor 488 (1:1000, gb2AF488, ChromoTek), goat anti-mouse IgG Alexa Fluor 488 (1:500, A-11001, Invitrogen), goat anti-rabbit IgG Alexa Fluor 594 (1:500, A-11037, Invitrogen), goat anti-mouse IgG (H + L) Alexa Fluor 647 (1:500, A21235, Life technology).

### Colony formation

For dox treatment, *TCHP* knockdown Huh-7 Cells were seeded at 400 cells/well in 6-well plates, and dox (500 ng/mL) was added to induce TCHP depletion. Cells were harvested after 14 days when macroscopic colonies became visible.

For alisertib treatment, 1000 cells/well were plated in 6-well plates with dox (500 ng/mL) to induce *TCHP* knockdown. After 5 days of dox pretreatment, 0.1 μM alisertib was added for 48 h of treatment. The drug-containing medium was then replaced with fresh dox-supplemented medium. Cells were cultured for a total of 10 days until macroscopic colonies formed and processed for analysis.

Cells were then gently washed with PBS and fixed with 2 mL of 4% PFA for 30–60 min at room temperature. After PBS washing, cells were stained with 1% crystal violet solution (w/v) for 30 min. Excess dye was removed under running water, and plates were air-dried before high-resolution scanning. Image J software was used for statistical analysis.

### Drug treatments

For the cell death assay, *TCHP* knockdown Huh-7 cells were incubated with 20 μM z-VAD (A1902, APExBIO) and 20 μM necrostatin-1 (A4213, APExBIO) separately. After 48 h of treatment, cells were then stained with propidium iodide (PI), and the proportion of PI-positive cells was quantified by flow cytometry.

For the evaluation of synergistic combination of *TCHP* knockdown and alisertib (T2241, TargetMol) in vitro, cells after 3 days of dox induction were incubated with 0.1 μM alisertib for 4 h. Cells were then fixed for the following immunofluorescence assay, and the relative fluorescence intensities of p-AURKA in treated cells were quantified.

For alisertib treatment in vivo, mice were treated with 15 mg/kg alisertib once a day for 6 days, and then treatment was stopped for 1 day.

For the CTG cell viability assay, cells were incubated with 0, 10, 20, 40, 80, 160, 320, 640, 1280 nM alisertib for 48 h after 5 days of dox pretreatment. The IC50 values of different treatments were calculated through Dose-response-Inhibition Nonlinear Regression analysis in GraphPad Prism 8.

### Expression and purification of recombinant proteins

His-GFP-tagged full-length TCHP and His-RFP-tagged full-length AURKA were expressed in BL21 (DE3) cells. *E. coli* were grown at 37 °C in TB until the OD at 600 nm reached 0.8. Expression was then induced with 0.5 mM IPTG at 16 °C for 16 h. The cells were harvested by centrifugation and washed with ice-cold PBS once. Pelleted cells were resuspended in high salt lysis buffer (20 mM Tris-HCl, pH=8.0, 500 mM NaCl, 20 mM imidazole, 1 mM DTT, protease inhibitor cocktail K1010). Resuspended cells were lysed by French press. Total cell lysates were pelleted by 35,000 x g centrifugation at 4 °C for 60 min. Packed columns with 5 mL nickel beads were used for the purification of His-tagged TCHP and AURKA. Finally, proteins were eluted with elution buffer (20 mM Tris-HCl, pH=8.0, 500 mM NaCl, 20 mM imidazole, 1 mM DTT, protease inhibitor cocktail K1010).

### Phase separation assay

To validate whether AURKA could incorporate into the liquid droplets formed by TCHP coacervation, purified TCHP-GFP was mixed with purified RFP-AURKA at indicated concentrations (150 mM NaCl, pH = 7.0, 5% Ficoll). The proteins were buffer-exchanged into 150 mM NaCl with 5% Ficoll to a final volume of 10 μL. Reactions were carried out in 1.5 mL Eppendorf tubes at 37 °C for 5 min. The samples were then transferred to a glass slide covered with a cover glass at room temperature. Samples were imaged on an Olympus confocal microscope with a 60× oil-immersion objective lens.

### Quantitative real-time PCR

Total RNA was isolated by using TRIzol reagent (15596018CN, Invitrogen), and complementary DNA was synthesized by using ABScript III RT Master Mix (RK20429, ABclonal). Quantitative polymerase chain reaction (qPCR) analysis was performed using the SYBR Green Fast qPCR Mix (RK21206, ABclonal). Data were recorded in the qPCR thermal cycler QuantStudio5 (Thermo Fisher Scientific). The primers used in this assay were as follows: *TCHP* (F, 5’-CAGGAGAATCTGTTGAAGCAGCG; R, 3’-AGCTCCTCTTGGATCTGCTGTG); *Tchp* (F, 5′-AACAGGAGAACCTGCTGAGGCA-3′; R, 5′-CTGTTGAGCTGCGCGTTGTACT-3′), and GAPDH (F, 5′-GTCTCCTCTGACTTCAACAGCG-3′; R, 5′-ACCACCCTGTTGCTGTAGCCAA-3′).

### Plasmids, shRNA design and cell culture

Human *TCHP* (NM_001143852.2) was cloned into the pLenti-EGFP-FLAG lentiviral vector to generate the Huh-7 cell line expressing GFP-FLAG-tagged TCHP. Mouse *Tchp* (NM_029992.3) was cloned into the Sleeping Beauty transposon pT3 vector to establish *Tchp*-overexpression in vivo model.

The shRNA targeting *TCHP* and *Tchp* were designed using The Genetic Perturbation Platform (GPP). The human *TCHP* shRNA sequence 5’-GGCAGAATGGAGCTCTAAA-3’ and 5’-GCGTTTCTTGAGACATCAGTA-3’ were used to generate *TCHP*-knockdown Huh-7 cell line. The following shRNA sequences were cloned into the Sleeping Beauty transposon pT2 vector:

Mouse *Tchp* shRNA#1, 5’-ATCAGCAGGAGGACCAGTTTC-3’;

Mouse *Tchp* shRNA#2, 5’-ATCAGCAGGAGGACCAGTTTC-3’;

Mouse *Tchp* shRNA#3, 5’-ATCAGCAGGAGGACCAGTTTC-3’;

Mouse *Tchp* shRNA#4, 5’-AGGAGGAGAAGAGGAAGATTC-3’.

The shRNAs targeting mouse *Tchp* were co-transfected with mouse *Tchp* expression vectors into HEK293T cells to validate their knockdown efficiency. Huh-7 (SCSP-526, Cell Bank of Chinese Academy of Sciences), HepG2 (ATCC), HEK293T (ATCC) cells were cultured in Dulbecco’s modified Eagle Medium (L110KJ, BasalMedia) supplemented with 10% fetal bovine serum (FSP500, ExCell Bio) and 100 IU/mL of penicillin/streptomycin (sv30010, HyClone). All cells were cultured at 37 °C in a humidified atmosphere with 5% CO_2_.

### Statistical analysis

All experiments were repeated at least three times. For quantification of tumor area in H&E staining sections, three random fields per sample were analyzed using ImageJ, with the mean area value representing the tumor burden. For cell proportion or area quantification, 3–5 randomly selected fields per group were analyzed using ImageJ software. For relative fluorescence intensity analysis, the average of 50 cells per group was calculated. Images were analyzed using Image J. Statistical analysis of the data was performed using GraphPad Prism 8. Data from triplicate biological experiments are presented as the mean ± SD. Statistical significance was tested using one-way ANOVA, two-way ANOVA or two-tailed Student’s *t* test, and *p* values are labeled in the figure panels with ns (non-significance, *p* > 0.05, *p < 0.05, **p < 0.01, and ***p < 0.001, ****p < 0.0001. *p* < 0.05 was considered statistically significant.

## Supplementary information

Supplementary Figs

uncropped original western blots

## Data Availability

The data are available from the corresponding author upon a reasonable request.
