# Supplementary material for: TCHP drives hepatocarcinogenesis through LLPS-mediated AURKA condensation and enables synergistic therapy
Source: Cell Death Dis. 2026 Apr 25;17(1):551. doi: 10.1038/s41419-026-08681-6 (PMC13247272; doi:10.1038/s41419-026-08681-6)
Supplement: Supplementary file 1 — Supplementary Figs [file 41419_2026_8681_MOESM1_ESM.pdf]

Supplementary figures for

TCHP drives hepatocarcinogenesis through LLPS-mediated AURKA condensation and enables synergistic therapy

Jingshi Li[1, †], Yan Li[1, †], Xilang Pan[1, †], Shu Wang[2, †], Zaisheng Lin[1, †], Ruida He[1], Suning Wang[1], Xin Huang[1], Jiaqi Li[3], Zhiwei Zhang[4], Liang Wang[5], Xiujuan Zhang[1, #], Xuxu Sun[2, #], Muqing Cao[1, #]

[1] Precision Research Center for Refractory Diseases, Shanghai Jiao Tong University Pioneer Research Institute for Molecular and Cell Therapies, Shanghai General Hospital, Shanghai Jiao Tong University School of Medicine, State Key Laboratory of Innovative Immunotherapy, School of Pharmaceutical Sciences, Shanghai Jiao Tong University, Shanghai, China; Department of Pathophysiology, College of Basic Medical Sciences, Shanghai Jiao Tong University, Shanghai, China.

[2] Department of Biochemistry and Molecular Cell Biology, State Key Laboratory of Systems Medicine for Cancer, Shanghai Key Laboratory for Tumor Microenvironment and Inflammation, Shanghai Jiao Tong University School of Medicine, Shanghai, China.

[3] Department of Gastroenterology, Children's Hospital of Soochow University, Suzhou, China.

[4] Department of Oncology, Affiliated Hospital of Hebei University of Engineering, Handan, China.

[5] School of Life Sciences, Jiangsu Normal University, Xuzhou, China.

[†] These authors share co-first authorship.

[#] To whom correspondence may be addressed: Xiujuan Zhang, Xuxu Sun, and Muqing Cao (Lead Contact). Email: zhangxj23@sjtu.edu.cn, xuxu.sun@shsmu.edu.cn, and muqingcao@sjtu.eud.cn (Lead Contact).

Authors' contributions: MC and XS conceived the concept and supervised the studies. JL, YL, ZL, RH, and ShW performed the animal experiments. XP performed bioinformatic analysis. JL, YL, XP, ShW, SW, XH, and ZL performed biochemical analysis, cell biology, imaging, and data analysis. ZZ, LW, and XZ analyzed data. JL, YL, XP, ShW, and ZL contributed equally to this study and thus are credited as co–first authors. MC, XS, JL, YL, XP, and ZL wrote the manuscript with input from all authors.

Conflicts of interest: The authors of this study declare that they do not have any conflicts of interest.

This PDF file includes:

Figures S1 to S9

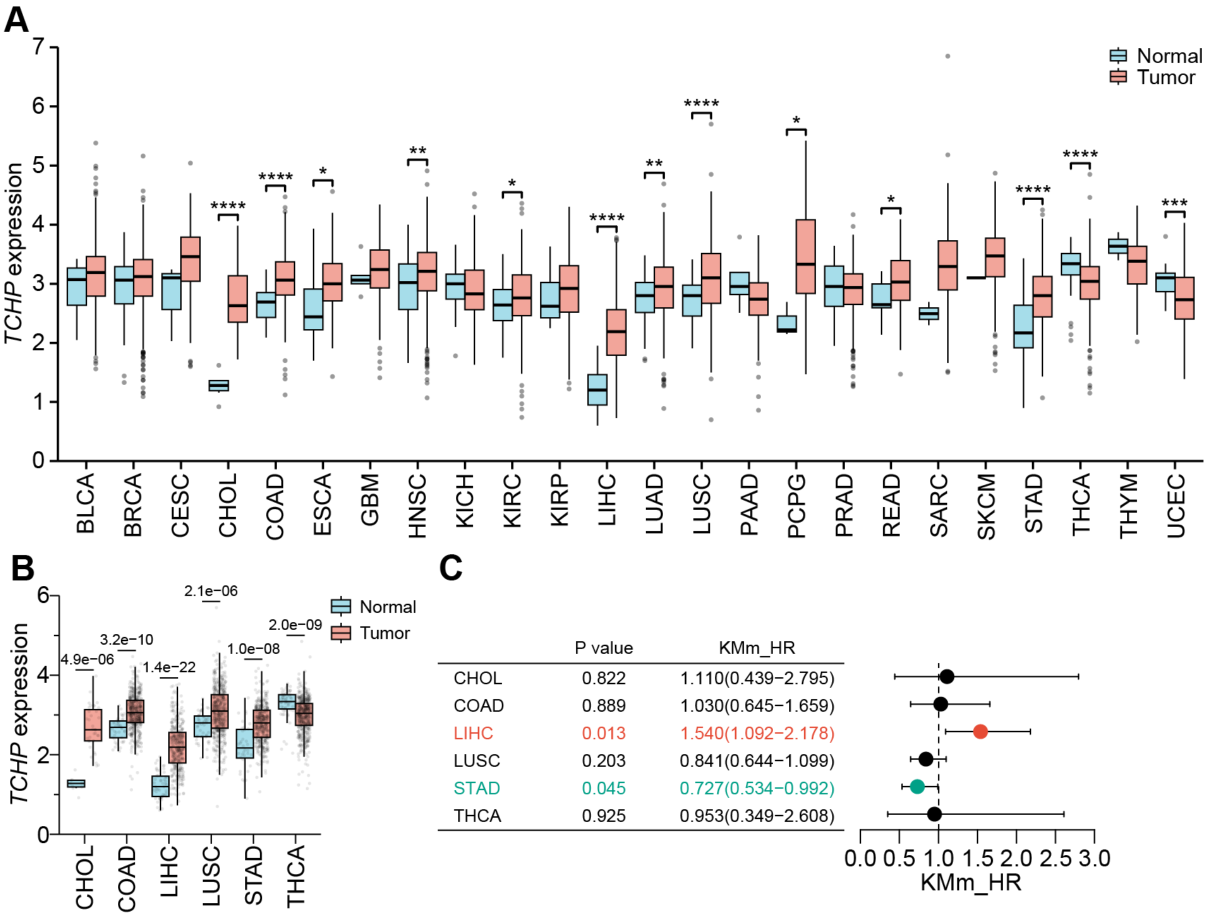

Fig. S1. Expression and prognostic analysis of *TCHP* in various cancers.

(A) Pan-cancer analysis of *TCHP* expression. (B) Analysis of *TCHP* expression in six cancer types exhibited the most significant differential *TCHP* expression. (C) Prognostic analysis of *TCHP* in six cancer types exhibited the most significant differential *TCHP* expression.

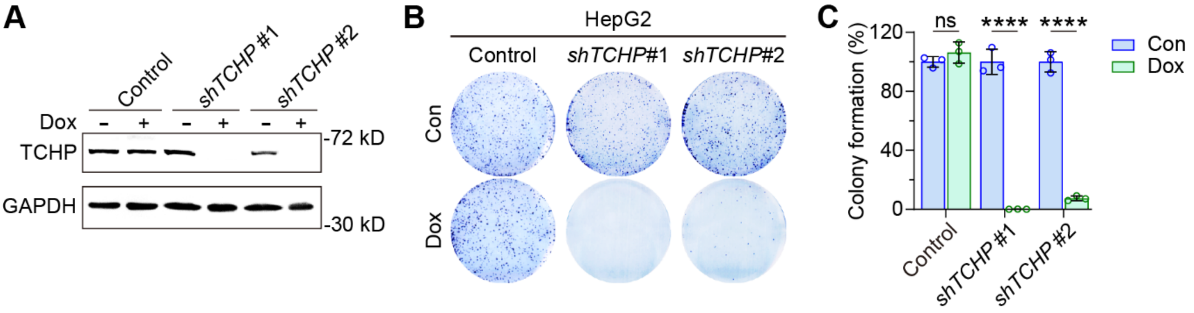

Fig. S2. *TCHP* knockdown leads to reduced colony formation in HepG2 cells.

(A) Western blot analysis of TCHP levels before and after dox treatment in control and *TCHP*-knockdown HepG2 cells. (B) After dox treatment, *TCHP*-knockdown HepG2 cells showed decreased average colony numbers in the plate colony formation assay. (C) Quantification of colony counts in (B). Data were presented as mean ± SD (n = 3; two-tailed unpaired Student's *t* test).

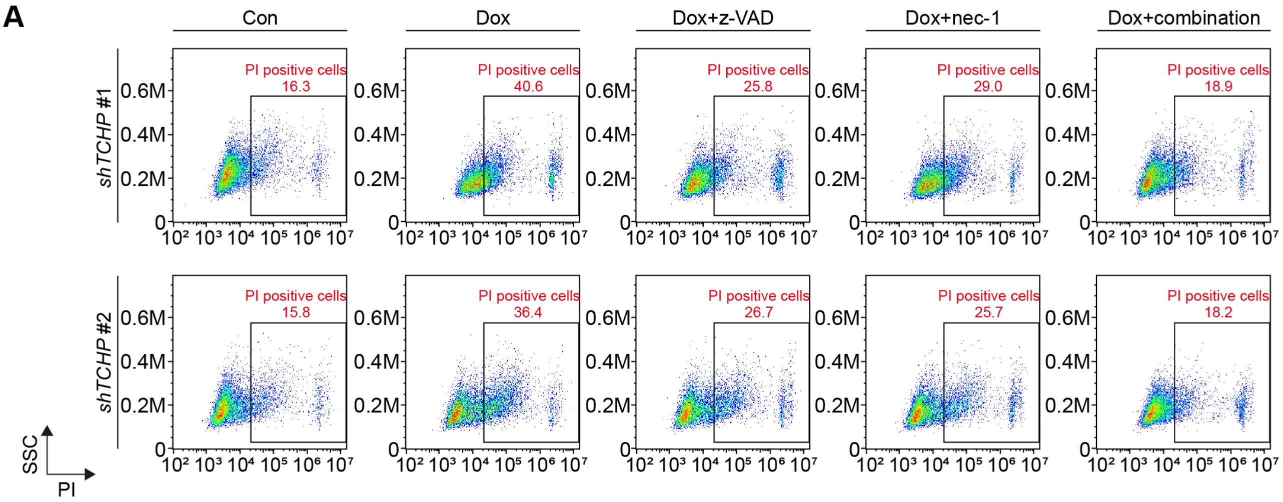

Fig. S3. *TCHP* knockdown induces cell death in liver cancer cells.

(A) Flow cytometry analysis of PI-positive cells in *TCHP*-knockdown Huh-7 cells with 20 μM z-VAD, 20 μM nec-1 and combination treatment.

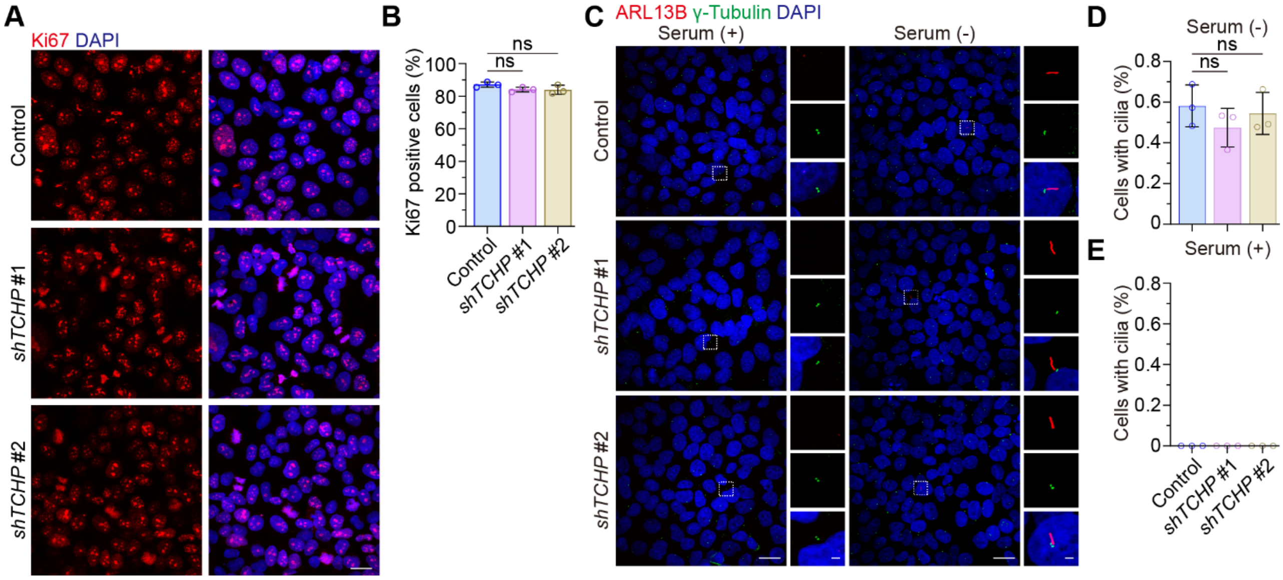

Fig. S4. *TCHP* knockdown in liver cancer cells does not induce G0/G1 cell cycle arrest or ciliogenesis.

(A) Representative immunofluorescence images of Ki67 (red) and DAPI (blue) in control and *TCHP*-knockdown Huh-7 cells. Scale bar = 20 μm. (B) Quantification of Ki67-positive cells in (A). Data were presented as mean ± SD (n = 3; one-way ANOVA with Dunnett's multiple comparisons test). (C) Representative immunofluorescence images of ARL13B (red), γ-tubulin (green) and DAPI (blue) in control and *TCHP*-knockdown Huh-7 cells under serum-starved and non-starved conditions. Scale bar = 20 μm. (D, E) Quantification of ciliated cells under non-starved (D) and serum-starved (E) conditions in (C). Data were presented as mean ± SD (n = 3; one-way ANOVA with Dunnett's multiple comparisons test).

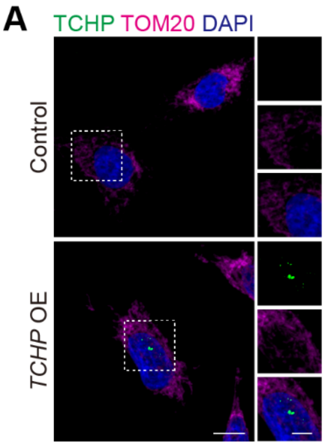

Fig. S5. TCHP does not colocalize with mitochondrial marker TOM20.

(A) Representative immunofluorescence images of TCHP (green), TOM20 (magenta) and DAPI (blue) in control and *TCHP*-overexpressed Huh-7 cells. Scale bar = 10 μm (left), 5 μm (right).

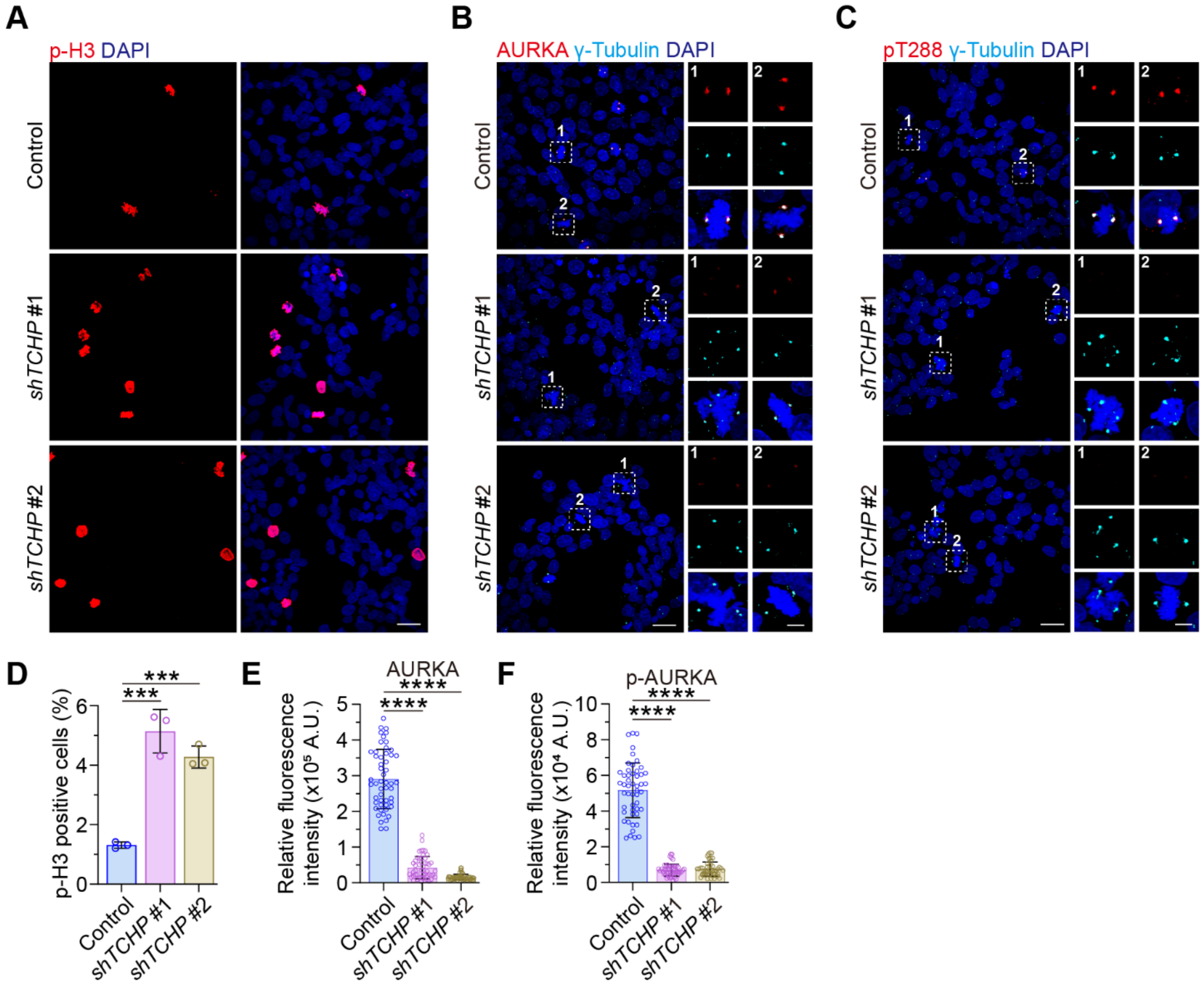

Fig. S6. *TCHP* knockdown induces the same phenotype in HepG2 cells.

(A) Representative immunofluorescence images of p-H3 (red) and DAPI (blue) in control and *TCHP*-knockdown HepG2 cells. Scale bar = 20 μm. (B) Representative immunofluorescence images of control and *TCHP*-knockdown cells stained with AURKA (red), γ-tubulin (cyan) and DAPI (blue). Scale bar = 20 μm (left), 5 μm (right). (C) Representative immunofluorescence images of p-AURKA (red), γ-tubulin (cyan) and DAPI (blue) in control and *TCHP*-knockdown HepG2 cells. Scale bar = 20 μm (left), 5 μm (right). (D) Quantification of p-H3 positive cells in (A). Data were presented as mean ± SD (n = 3; one-way ANOVA with Dunnett's multiple comparisons test). (E, F) Relative fluorescence intensity quantification of AURKA (E) and p-AURKA (F) in (B), (C). Data were presented as mean ± SD (average of 50 cells per group; one-way ANOVA with Dunnett's multiple comparisons test).

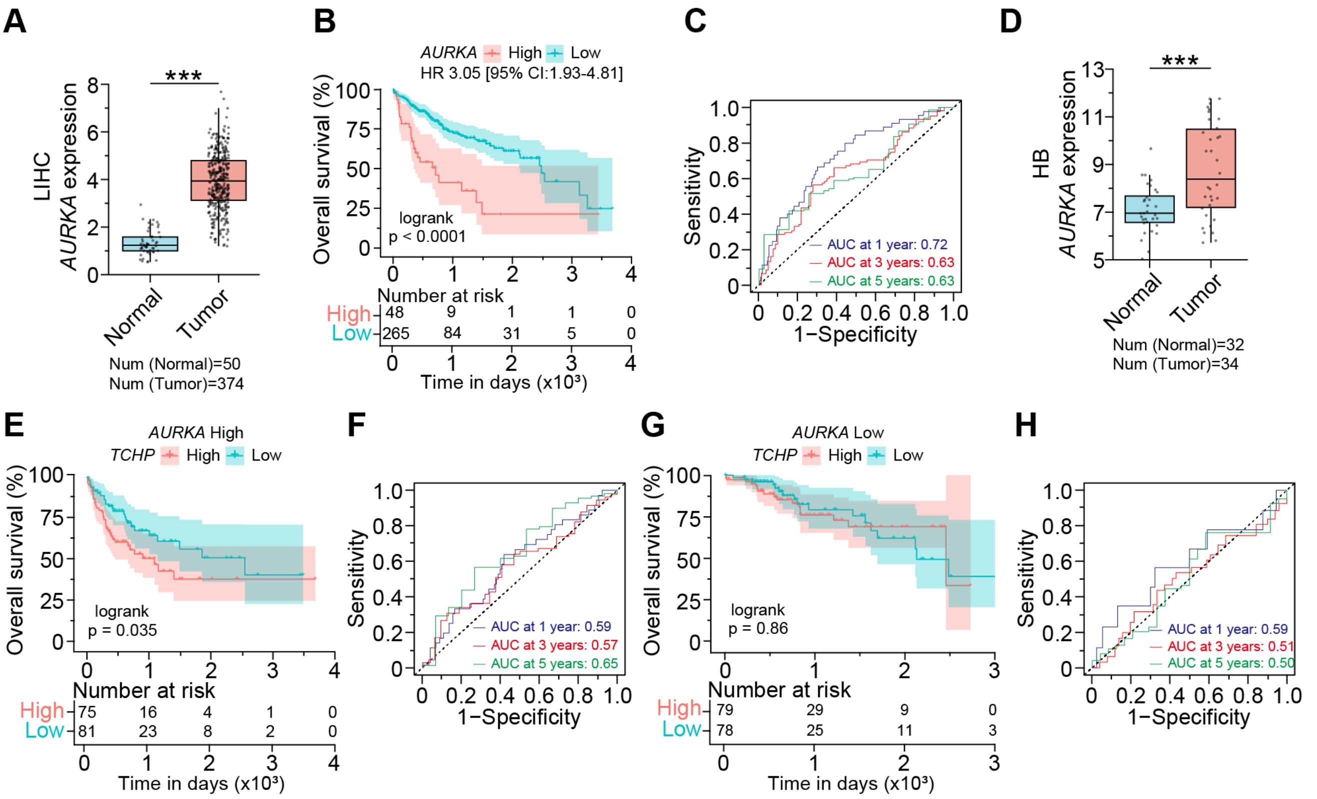

Fig. S7. Patients with concurrent high *TCHP* and *AURKA* expression showed the poorest prognosis.

(A) Comparison of *AURKA* expression between normal (n = 50) and tumor (n = 374) tissues in the TCGA-LIHC cohort. (B) Kaplan-Meier curves of overall survival based on *AURKA* expression in 444 LIHC patients (Log-rank test, hazard ratio = 3.05, p < 0.0001). (C) ROC curve analysis was performed to describe the accuracy of the scores for predicting 1-, 3-, and 5-year overall survival in the TCGA-LIHC cohort. (D) Comparison of *AURKA* expression between normal (n = 32) and tumor (n = 34) tissues in the R2-HB cohort. (E-H) Cox proportional hazards analysis was performed to evaluate the combined effect of *TCHP* and *AURKA* expression levels on the overall survival of LIHC patient cohort from the TCGA repository (E, G). ROC analysis was performed to describe the accuracy of the scores for predicting 1-, 3-, and 5-year overall survival in TCGA-LIHC patients (F, H).

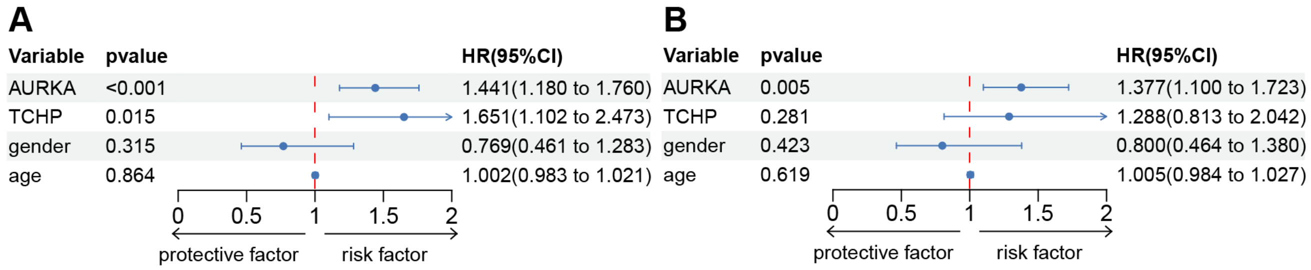

Fig. S8. Cox regression analysis of *TCHP* and *AURKA* in LIHC.

(A-B) Univariate Cox regression (A) and multivariate Cox regression (B) analysis of *TCHP* and *AURKA*.

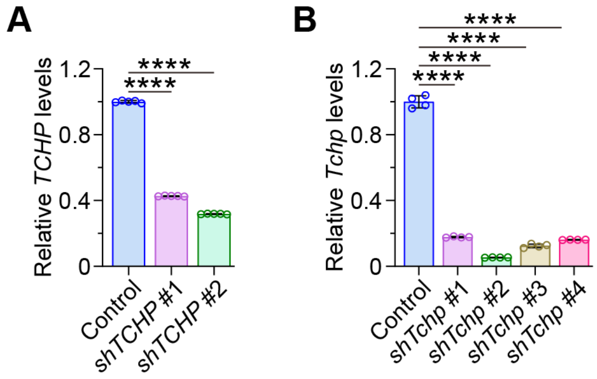

Fig. S9. The knockdown efficiency of shRNAs targeting human *TCHP* and mouse *Tchp*.

(A) RT-qPCR demonstrating reduced expression of *TCHP* in *TCHP*-knockdown Huh-7 cells. Data were presented as mean ± SD (n = 5; one-way ANOVA with Dunnett's multiple comparisons test). (B) RT-qPCR demonstrating reduced expression of *Tchp* in *Tchp*-knockdown HEK293T cells. Data were presented as mean ± SD (n = 4; one-way ANOVA with Dunnett's multiple comparisons test).
