# Supplementary material for: TCHP drives hepatocarcinogenesis through LLPS-mediated AURKA condensation and enables synergistic therapy
Source: Cell Death Dis. 2026 Apr 25;17(1):551. doi: 10.1038/s41419-026-08681-6 (PMC13247272; doi:10.1038/s41419-026-08681-6)

Original western blots for Figure 2

Figure 2A

TCHP

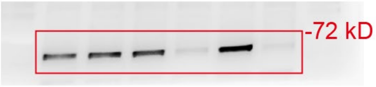

GAPDH

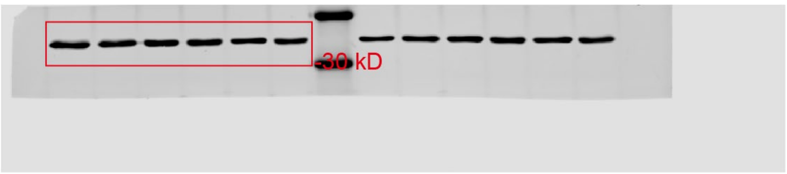

Original western blots for Figure 3

Figure 3C

TCHP

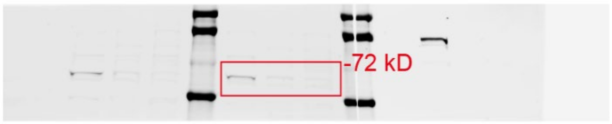

GAPDH

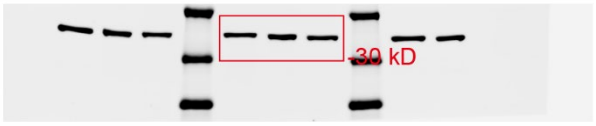

Original western blots for Figure 4

Figure 4D

FLAG

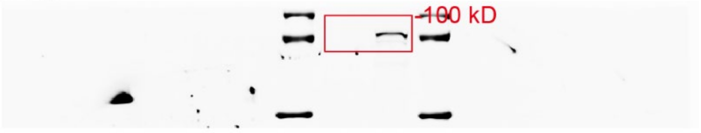

GAPDH

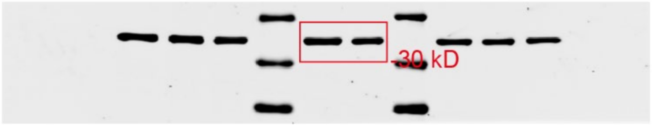

Original western blots for Figure S2

Figure S2A

TCHP

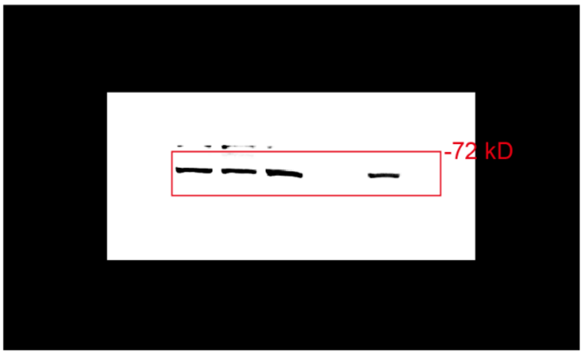

GAPDH

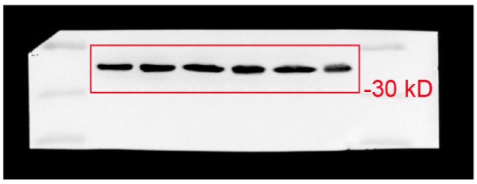

Supplement: Supplementary file 2 — uncropped original western blots [file 41419_2026_8681_MOESM2_ESM.pdf]
